# Clinical significance of serum T helper 1/T helper 2 cytokine shift in patients with non-small cell lung cancer

**DOI:** 10.3892/ol.2014.2391

**Published:** 2014-07-29

**Authors:** JUN LI, ZHENHUA WANG, KAI MAO, XIXI GUO

**Affiliations:** Department of Thoracic Surgical Oncology, Xinxiang Central Hospital, Xinxiang, Henan 453000, P.R. China

**Keywords:** non-small cell lung cancer, postoperative survival time, relapse, T helper 1/T helper 2 cytokine shift

## Abstract

The aim of this study was to explore the T helper 1 (Th1)/Th2 cytokine shift and its clinical significance in the peripheral blood and tumor tissues of non-small cell lung cancer (NSCLC) patients. In total, 124 NSCLC patients who were admitted to Xinxiang Central Hospital were selected, along with 124 healthy individuals undergoing physical examination at the same hospital during this period (as controls). ELISA was conducted to detect the Th1 and Th2 cytokine levels in the peripheral blood of patients in the two groups prior to and following radical surgery treatment. In addition, the Th1 and Th2 cytokine levels in the peripheral blood of the observation group were measured following surgery to analyze the correlation between relapse and survival. Compared with the control group, interleukin 4 (IL-4) and IL-10 concentrations in the peripheral blood of the observation group, prior to and following surgery, were significantly higher, whilst IL-2 and interferon-γ (INF-γ) concentrations were significantly lower (P<0.05). In the observation group, the IL-4 and IL-10 concentrations were significantly decreased following surgery, as compared with prior to surgery (P<0.05), whilst the IL-2 and INF-γ concentrations increased significantly (P<0.05). The one- and three-year cumulative relapse frequencies of patients with postoperative IL-4 abnormalities were significantly increased compared with those in patients with normal IL-4 levels following surgery (P<0.05), and the median survival time and survival rate significantly decreased in patients with postoperative IL-4 abnormalities (P<0.05). In terms of the three-year cumulative relapse rate, median survival time, and one- and three-year cumulative survival rate, patients with postoperative IL-2, IL-10 and INF-γ level abnormalities did not present any statistical significance compared with those without such abnormalities (P>0.05). In conclusion, Th2 cytokines dominate the peripheral blood of NSCLC patients and radical surgery treatment may improve the Th1/Th2 shift in patients. Furthermore, postoperative IL-4 levels were observed to correlate with relapse and the survival rate of patients; therefore, IL-4 may be considered as an auxiliary in the postoperative diagnosis during clinical practice.

## Introduction

Lung cancer is one of the most commonly observed malignancies in clinical diagnosis. In total, ~85% of lung cancer cases are diagnosed as non-small cell lung cancer (NSCLC). Due to environmental deterioration and changes in lifestyle, the incidence rate of NSCLC has significantly increased, becoming one of the predominant diseases to threaten human health (1,2). Radical surgery is commonly used to treat NSCLC; however, relapse frequently occurs following surgery, which reduces the postoperative survival time of patients (3). Causes of relapse in NSCLC may include the incomplete resection of the tumor and the inhibition of the human immune system, which may enable tumor cells to avoid immune killing (4). CD4^+^ T lymphocytes can be divided into T helper 1 (Th1) and Th2 cell subgroups, according to the secreted cytokine types. The Th1 subgroup releases interleukin 2 (IL-2) and interferon-γ (INF-γ), which has antitumor effects in the human body. The Th2 subgroup predominantly releases IL-4 and IL-10, which are involved in the inhibition of the immune system, preventing the human body from killing tumor cells (5,6).

In several studies, the Th1/Th2 immunological balance in tumor patients has been shown to be significant in tumorigenesis, development and relapse (7,8). A number of studies have also revealed that in breast, gastric and lung cancer patients, the immunological balance of Th1 and Th2 in the peripheral blood is altered, with the human body maintaining a Th2-dominant shift. The immunodominance of Th2 prevents the patient from effectively killing the tumor cells that have survived incomplete resection, which eventually results in relapse (9–11). Studies have indicated that when Th2 cytokines are dominant in the peripheral blood of NSCLC patients, the patient’s immunity is in a state of immune tolerance (12,13). However, few studies have investigated the changes in the levels of Th1 and Th2 cytokines in the peripheral blood following surgery, as well as the correlation between such changes and relapse or survival. Therefore, the current study began by analyzing the changes in the levels of Th1 and Th2 cytokines in NSCLC patients prior to and following surgery. In addition, the correlation between cytokine levels and patient relapse and survival was explored. The aim of this study was to investigate the serum Th1/Th2 cytokine shift and its clinical significance in the tumor tissues of patients with NSCLC.

## Patients and methods

### Patient presentation

A total of 124 patients with NSCLC, who were admitted to Xinxiang Central Hospital (Xinxiang, China) between June 2010 and June 2013, were selected for this study. All patients underwent radical surgery treatment and were diagnosed pathologically with NSCLC. All patients did not receive chemotherapy, radiotherapy or immune enhancer treatment prior to or following surgery. The hepatorenal functions of patients were normal and patients had no general infectious diseases. Of the 124 patients, 82 were male and 42 were female, with an average age of 60.25±10.01 years (range, 40–75 years). In terms of pathological types, there were 43 cases of squamous cell carcinoma, 60 cases of adenocarcinoma and 21 cases of mixed type. For TNM staging (3), there were 50 cases of stage IIa and 74 cases of stage IIb. Concomitantly, 124 healthy people undergoing physical examination at the same hospital during this period were selected as controls, including 90 males and 34 females, with an average age of 58.65±11.42 years (range, 41–69 years). The comparison between the control and observation groups with respect to gender and age range did not show statistical significance, exhibiting a certain comparability (P>0.05). This study was conducted in accordance with the declaration of Helsinki and with approval from the ethics committee of Xinxiang Central Hospital (Xinxiang, China). Written informed consent was obtained from all participants.

### Detection of cytokines

In the observation group, 5 ml of venous blood was obtained 10 days prior to and 10 days following surgery, and was not processed with anticoagulation. Following the stratification of blood by 30 min of quiescence at 4°C, the blood was centrifuged at 5,000 × g for 5 min and the serum was extracted. Serum of the control group was collected using the same approach. Samples were frozen at −80°C for further use. An ELISA kit (R&D Systems Inc., Minneapolis, MN, USA) was used to measure IL-2, IL-4, IL-10 and INF-γ levels in the serum. During the assay, standard curves were established for all four cytokines, and the corresponding cytokine concentration was calculated according to the standard curve. The assays were conducted according to the manufacturer’s instructions. All experiments for each sample were performed in triplicate. The normal ranges for the four cytokines were as follows: IL-2, 1.5–3.6 mg/ml; IL-4, 1.8–2.9 mg/ml; IL-10, 2.5-6-4.6 mg/ml; and IFN-γ, 7.3–16.5 mg/ml. Cytokine levels outside of these ranges were considered to be abnormal.

### Statistical analysis

All data were analyzed using SPSS 17.0 (SPSS, Inc., Chicago, IL, USA), and the data are presented as the mean ± standard deviation. Comparisons of measurement data were assessed by Student’s t-test, and comparisons of count data were assessed using Pearson’s χ^2^ test. Comparisons between cumulative relapse frequencies and the cumulative survival rate were assessed by Kaplan-Meier and log-rank tests. P<0.05 was considered to indicate a statistically significant difference.

## Results

### Comparison between the Th1/Th2 cytokine levels of two groups prior to and following surgery

**Figure 1:**
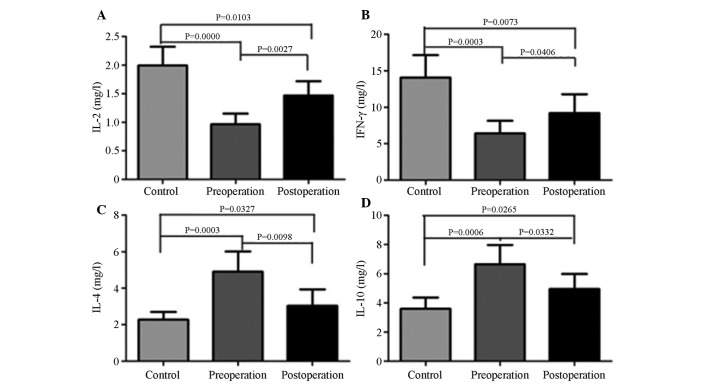
Comparison between the Th1/Th2 cytokine levels in the control group and observation group (pre- and postoperatively). (A) IL-12, (B) INF-γ, (C) IL-4 and (D) IL-10 levels observed in the control group (pre- and postoperatively). Th, T helper; IL, interleukin; INF-γ, interferon-γ.

Compared with IL-4 (2.28±0.27 mg/ml) and IL-10 (3.60±0.32 mg/ml) levels in the control group, the IL-4 (4.90±0.46 mg/ml) and IL-10 (6.65±0.54 mg/ml) concentrations in the peripheral blood of the observation group prior to surgery were significantly higher (P<0.05) (Fig. 1). Following surgery, the IL-4 (3.03±0.37 mg/ml) and IL-10 (4.96±0.42 mg/ml) concentrations in the peripheral blood of the observation group had decreased significantly compared with the levels prior to surgery (P<0.05), but remained significantly higher than those in the control group (P<0.05).

Compared with the IL-2 (2.00±0.13 mg/ml) and INF-γ (14.07±1.25 mg/ml) levels in the control group, the IL-2 (0.97±0.08 mg/ml) and INF-γ (6.40±0.71 mg/ml) levels in the peripheral blood of the observation group prior to surgery were significantly lower (P<0.05). Additionally, following surgery, the IL-2 (1.47±0.10 mg/ml) and INF-γ (9.21±0.91 mg/ml) levels in the peripheral blood of the observation group had increased significantly compared with the levels prior to surgery (P<0.05), but remained significantly lower than those in the control group (P<0.05).

### Correlation between various cytokines and patient relapse

**Figure 2:**
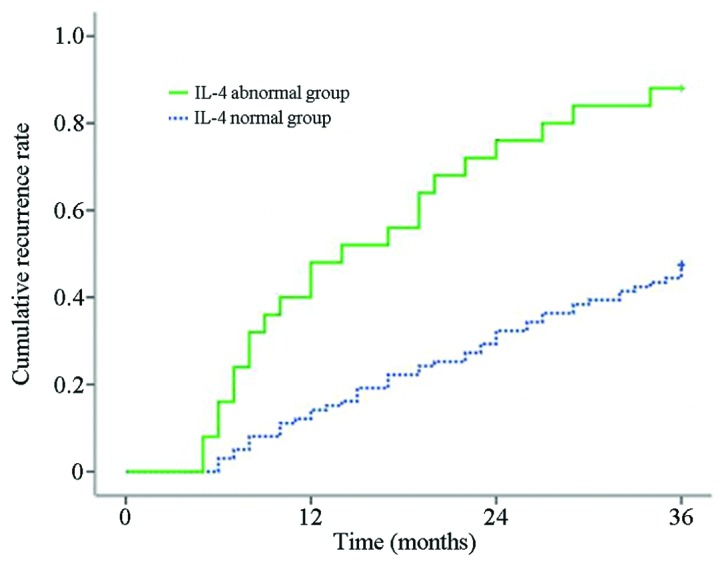
Correlation between IL-4 levels and postoperative recurrence in non-small cell lung cancer patients. IL-4, interleukin-4.

As shown in Table I and Fig. 2, the one- and three-year cumulative relapse rates of patients with postoperative IL-4 abnormalities were higher than those of patients without postoperative IL-4 abnormalities, and the difference showed statistical significance (P<0.05). Furthermore, the one-and three-year cumulative relapse rates of patients with postoperative IL-2, IL-10 and INF-γ abnormalities were not significantly different compared with those of patients without such abnormalities (P>0.05).

### Correlation between various cytokines and patient postoperative survival

As shown in Table II, the median survival time and the cumulative survival rate of patients with postoperative IL-2, INF-γ and IL-10 level abnormalities were not significantly different compared with those of patients without these abnormalities (P>0.05). However, the median survival time and cumulative survival rate of patients with abnormal postoperative IL-4 levels were significantly lower compared with those of patients with normal postoperative IL-4 levels (P<0.05).

## Discussion

NSCLC is one of the most commonly observed lung cancers in clinical practice, with morbidity and mortality exhibiting a significant increasing trend, and with a poor postoperative prognosis. NSCLC has become one of the predominant causes of cancer-related mortalities in a number of countries (14). In recent years, with the progression of tumor immunology studies, Th1 cytokines have been found to dominate the peripheral blood of numerous tumor patients (15). However, certain studies have also identified a Th2 cytokine shift in tumor cells. This phenomenon has been closely associated with clinical stages and postoperative effects in patients (15).

The present study analyzed the levels of Th1 and Th2 cytokine expression in the peripheral blood of NSCLC patients. The results demonstrated that, compared with the healthy controls, the levels of Th1 cytokines, IL-2 and INF-γ, in the peripheral blood of NSCLC patients significantly decreased. However, the levels of Th2 cytokines, IL-4 and IL-10, significantly increased, indicating that the NSCLC patients exhibited an evident Th2 cytokine shift. This finding is consistent with the majority of existing studies (13,16). Notably, following the resection of NSCLC, the Th1 cytokines, IL-2 and INF-γ, in the peripheral blood increased significantly compared with the levels prior to surgery, while levels of the Th2 cytokines, IL-4 and IL-10, decreased. The Th1/Th2 cytokine shift was improved to a certain degree, but did not reach the normal level, indicating that the resection of the tumor may help to combat patient immunosuppression.

For NSCLC patients, the relapse rate is relatively high and the survival time is shorter than that for other cancer types (17,18). The main causes of this phenomenon not only include incomplete resection, but also closely correlate with the inhibition of the patient’s immune system (17). The present study demonstrated that although surgery may improve immunosuppression in patients, the effects were limited. For certain patients, the Th1 and Th2 cytokine levels remained abnormal following the resection. However, if the Th1 and Th2 cytokine levels are normal, even if residual tumor cells exist, the immune system may kill these remaining tumor cells and suppress the relapse of the disease. By contrast, whilst in a state of immunosuppression, the body cannot kill the tumor cells effectively and, thus, tumor relapse occurs.

The current study initially analyzed the correlation between Th1 and Th2 cytokine abnormalities and patient relapse. Patients with postoperative IL-4 abnormalities were found to exhibit a significantly shorter survival time, indicating that IL-4 levels are significant in the postoperative survival of NSCLC patients. However, the postoperative IL-2, IL-10 and INF-γ levels did not exhibit a marked impact on relapse. This finding has also been verified among breast cancer patients (19). The present analysis of patient survival time demonstrated that NSCLC patients with postoperative IL-4 level abnormalities exhibit a decreased median survival time and survival rate. This finding corresponded with the relapse rate of patients with postoperative IL-4 level abnormalities. The reasoning behind the abnormally high postoperative expression levels of IL-4 leading to reduced relapse and survival rates may be that IL-4 induces the differentiation of Th1 into Th2 and therefore, the lymphocyte activation was inhibited. IL-4 may also upregulate the antiapoptotic protein level, thereby reducing the killing ability towards tumor cells (20–22).

Notably, in the current study, no statistically significant difference was identified between the patient relapse and survival rates in patients with postoperative IL-2, IL-10 and INF-γ abnormalities. However, this may be attributed to the limited sample size. In addition, the present study adopted a one-way analysis of variance, but the tumor relapse and survival rates were influenced by multiple factors. Therefore, the correlation between these cytokines, tumor relapse and the survival rate should be analyzed based on multiple factors.

Furthermore, Th2 cytokines evidently dominated the peripheral blood of NSCLC patients, and surgery greatly improved this Th2 cytokine shift. The abnormal expression of IL-4 levels in the peripheral blood following surgery was also found to inversely correlate with patient relapse and survival rates. In conclusion, this study provides guidance regarding postoperative immune intervention in NSCLC patients.

## Figures and Tables

**Table I:** Comparison between the postoperative cumulative relapse rates of patients with varying cytokine levels.

		Cumulative relapse rate, %			
				
Cytokine	Cases, n	1-year	3-year	Log-rank	P-value^a^
IL-2					
Normal	91	19.78	42.96	2.456	0.147
Abnormal	33	24.24	42.42		
INF-γ					
Normal	82	17.07	50.00	0.063	0.802
Abnormal	42	21.43	52.38		
IL-4					
Normal	99	15.15	33.33	5.119	0.001^a^
Abnormal	25	40.00	72.00		
IL-10					
Normal	102	25.49	54.90	1.157	0.307
Abnormal	22	31.82	50.00		

a P<0.05, vs. control group.

IL; interleukin; INF-γ, interferon-γ.

**Table II:** Correlation between the various cytokine levels and postoperative survival time in patients with non-small cell lung cancer.

Cytokine	Cases, n	Median survival time (range), months	Cumulative survival rate, %		Log-rank	P-value^a^

			1-yea	r 3-year		
IL-2						
Normal	91	26 (13.34–31.35)	84.84	48.48	0.078	0.801
Abnormal	33	22 (9.56–35.21)	76.92	52.75		
INF-γ						
Normal	82	28 (16.49–36.14)	85.37	64.63	1.386	0.219
Abnormal	42	24 (12.56–30.87)	73.81	57.14		
IL-4						
Normal	99	26 (18.55–34.49)	90.90	78.79	4.487	0.021^a^
Abnormal	25	18 (7.51–25.39)	76.00	44.00		
IL-10						
Normal	102	28 (18.19–32.33)	73.53	46.08	1.987	0.115
Abnormal	22	26 (15.12–35.89)	68.18	40.91		

a P<0.05, vs. control group.

IL, interleukin; INF-γ, interferon-γ.
